# The More Insufficient, the More Avoidance? Cognitive and Affective Factors that Relates to Information Behaviours in Acute Risks

**DOI:** 10.3389/fpsyg.2021.730068

**Published:** 2021-09-24

**Authors:** Shuguang Zhao, Yiming Liu

**Affiliations:** ^1^School of Journalism and Communication, Nanjing University, Nanjing, China; ^2^Zijin Media Research, Nanjing University, Nanjing, China

**Keywords:** information seeking, information avoidance, risk communication, health communication, public health emergency, COVID-19, China

## Abstract

This study examines the relationship between cognitive and affective factors and people's information-seeking and -avoiding behaviours in acute risks with a 1,946-sample online survey conducted in February 2020, during the outbreak of the COVID-19 pandemic in mainland China. Multiple linear regression analysis showed that perceived information insufficiency correlates negatively with information-seeking behaviour and there was an inverted U-shaped relationship between information insufficiency and avoidance behaviour. As for the risk-related cognitive factors, information seeking increases as perceived severity of risks rises, while information avoiding increases as perceived susceptibility rises. Perceived response efficacy positively correlates with information-seeking and negatively with information-avoidance behaviours. Preliminary results also indicated that different affective factors relate to information-seeking and avoidance behaviours differently.

## Introduction

As of August 17, 2021, the COVID-19 pandemic has seen more than 208 million cases worldwide and over 4 million deaths (CSSE, 2020). The World Health Organisation (World Health Organization, 2020a) declared COVID-19 a Public Health Emergency of International Concern (PHEIC) in January 2020 and a pandemic in March 2020 (World Health Organization, 2020b). PHEICs are extraordinary events that are “determined to constitute a public health risk to other States through the international spread of disease and potentially require a coordinated international response” (World Health Organization, 2008, p. 9). The sudden and global impact of COVID-19 led many people to seek information. Google trends data show that “COVID-19” was the most-searched keyword worldwide in March and April 2020 (Google Trends, 2020). This indicates the importance of information availability during a public health crisis, but it foregrounds the need to ensure the proper management of a massive flow of risk-related information on the Internet and the 24-h news cycle. During the COVID-19 outbreak, people around the world are pressured to seek information about the spread of the virus and potential preventative measures at the governmental and individual level. As most people were asked to stay at home to prevent the virus's spread, most information-searching behaviours occurred online.

Previous research established various cognitive models explaining what drives information seeking behaviour. Their key hypotheses are based on cognitive processing models, including the Heuristic-Systematic Model (HSM), which has an information-oriented perspective (Chaiken, 1980), or the Extended Parallel Processing Model (EPPM) (Witte, 1994) with a risk-oriented perspective. Recent studies integrate the prediction model by incorporating cognitive processing and the affective dimension (Griffin et al., 2008; Ferrer et al., 2015). As intentional information avoidance was observed, researchers tried to enhance the model's generalizability by applying it to explain information-avoidance behaviour in risks. This research will compare how the cognitive factors and affective factors in these models correlate with the information seeking and avoidance behaviours under risks. It will also contribute to the understanding of the motives of people's information behaviours under the sudden occurrence of acute risks, which was less studied compared to the behaviours concerning chronic risks.

## Literature Review

### Risk Information Seeking and Avoidance

Risk is concerned with the uncertainty caused by an event's potentially undesirable consequences (ISO, ISO). Such uncertainty is concerned with the deficiency of information or knowledge to figure out the causes, possibilities, and consequences of the event. Barsevick and Johnson (1990, pp. 3–4) defined information-seeking behaviour as “actions used to obtain knowledge of a specific event or situation,” In communication research, information-seeking behaviour is defined as the purposive and active search for information which requires a certain level of effort and intensity (Yang et al., 2014). As such, information-seeking behaviour emphasises active and purposive behaviour, rather than passive media-scanning (Kelly et al., 2010). The sudden and novel risks brought by PHEICs are more salient in terms of their high severity of impact and low familiarity, requiring more public awareness to seek more risk-related information.

Information avoidance, described as “denial, blunting, or repression” (Lambert and Loiselle, 2007, p. 1009), refers to an individual's choice to divert attention from the information. According to Maslow (1963), the word “knowing” is related to the sense of “domination, mastery, control” and the fear of knowing stems from defensive instincts. Such defensive response applies to individuals' self-recognition and their perception of the environment. While an epidemic poses an acute threat to society, prolonged risk messages may overwhelm people, especially in light of massive and contradictory information circulating *via* various information channels, People may hide from distressing and disappointing news reports, and feel meaningless and powerless because high degrees of uncertainty make individual efforts seem senseless. As Case et al. (2005, p. 359) stated, “Avoiding information is closely linked to feelings of anxiety and fear as well as to other cognitive and emotional variables like perceptions of treatment efficacy, self-efficacy, and locus of control.tendencies towards fatalism and avoidance can short circuit any information seeking at all.”

Previous research has investigated the factors affecting people's information seeking behaviour under risks. Based on the Risk Information Seeking and Processing (RISP) model (Griffin et al., 1999) and other information behaviour prediction models, Kahlor (2010) proposed a comprehensive theoretical model, the Planned Risk Information Seeking Model (PRISM), which aims to explain the information seeking behaviour under risks. Further, they also proposed the Planned Risk Information Avoidance (PRIA) model, that illustrate the links between cognitive as well as affective factor and information avoidance behaviour under risks. (Deline and Kahlor, 2019). For information behaviour for risks, the cognitive factors could be subdivided into information-oriented motivators and risk-oriented motivators. The information-oriented motivators comply with the basic assumption that people make economy-minded decisions on information processing strategies by maximising information sufficiency with the fewest cognitive resources (Eagly and Chaiken, 1993). Variables such as cognitive load and need for closure in the PRISM and PRIA models reveals such information sufficiency principle. The risk-oriented motivators emphasise the influence of fear induced by risks, in which people weigh the severity of the risks against their ability to cope with it. Variables such as risk perceptions and perceived behavioural control in the PRISM and PRIA models reveals such fear-control principle. Affective responses are less considered to be the predictor of information-related behaviour and but more often considered to be antecedents or consequences of the above-mentioned cognitive factors. The information-oriented motivators and risk-oriented motivators apply to different scenarios. The information sufficiency principle could account for general risk-related information-seeking behaviour, as it assumes that people satisfy their cognitive need for information and assess risk-related information rationally, with a specific goal in mind, such as having sufficient information to act. In contrast, the fear-control principle might account for information behaviour under salient threats or hazards, especially in the situation where the information sufficiency principle may overestimate human rationality and efficiency in extreme cases (Rice and Atkin, 2012). The information behaviour prediction models comprehensively explain how cognitive and attitudinal factors are related to people's information-seeking and -avoiding behaviours. while differences underlying the two principles need to be compared.

### Cognitive Factors That Related to Information-Related Behaviour

#### Perceived Information Insufficiency

Perceived information sufficiency in PRISM, similar to concept of the need for closure in the later model PRIA, refers to the amount of information or knowledge that individuals subjectively think they require to have a satisfactory judgment confidence level (Griffin et al., 2004b). Accordingly, perceived information insufficiency identifies the gaps between individuals' sufficiency thresholds and their actual knowledge (Griffin et al., 2004a) that is, it measures discrepancies between individuals' actual and desired judgmental confidence. It focuses on the need for information and assumes people choose different information-processing strategies according to the sufficiency principle, “people will exert whatever effort is required to attain a “sufficient” degree of information to make a choice,” (Eagly and Chaiken, 1993, p. 330). When people perceive they lack actionable knowledge regarding concerns, they are more likely to process issue-related information in a systematic and effortful way. Multiple models indicate that such discrepancy motivates people to seek and process information in active and systematic ways, and therefore suggest a positive relationship between the cognitive need for information sufficiency and information-seeking behaviour (Griffin et al., 2004a; Kahlor et al., 2006; Kahlor, 2010). The information behaviour prediction models emphasise the prominent role played by people's desire for information sufficiency. For example, the RISP model suggests perceived information insufficiency and subjective information-related norms drive people's risk-related information-seeking behaviour (Yang et al., 2014). In that way, information-seeking behaviour is continuous and goal-oriented (Gutteling and de Vries, 2017).

Due to limited cognitive capacity, when individuals feel their need for information sufficiency has been satisfied, they allocate less time and effort to reaching out for new information. In such situations, people may avoid exposure to more relevant information and pay selective attention to new information such as obtaining it from limited sources or thinking less critically about the information they encounter (Kahlor et al., 2006). This suggests that perceived information insufficiency may positively correlate with people's information avoidance behaviour. For risk-related information, individuals may be more likely to maintain certain degree of uncertainty because of the overload brought by the undesirable risk-related information (Yang et al., 2014). Yang and Kahlor (2013) found perceived information insufficiency were not a significant predictor of information-avoidance behaviour concerning the chronic risk, the climate change issue. They suggested that, at least in some contexts, the driving force to seeking or avoiding information may be for reasons other than information sufficiency. We therefore propose that the principle of information sufficiency still plays a role in motivating people's information seeking in acute risks when their information needs about the novel threats are urgent. At the same time, as their confidence in the information sufficiency increases, individuals are more inclined to avoid the undesirable information related to the risks.

#### Perceived Risk

Risk perception originates from the protection motivation theory (Rogers, 1975). It posits that threat-related messages stimulate people's motivation to protect themselves through two channels: threat appraisal (gauging the severity of a situation) and coping appraisal (assessing the capability of one's response to the situation). They focus on people's cognitive processing messages relative to risks and examines how they react to their perceptions. This approach views information-seeking and -avoidance behaviour as a response to fear aroused by perceived threats.

Witte (1994) further illustrates such cognitive process in the Extended Parallel Model which sees both the success and failure of the fear appeal as possible behavioural mechanisms. The model proposes that people will adopt different information-processing strategies depending on their cognitive appraisals of messages; specifically, how they balance their risk perception and efficacy beliefs (Miles et al., 2008). Risk perception is the “appraisal of threats” and efficacy beliefs are the “appraisal of the efficacy of the recommended response” (Witte et al., 2001, p. 24). First, threat appraisal determines whether fear is aroused when an individual evaluates the seriousness of a threat and its potential impact. Second, the aroused fears encourage individuals to respond to or control their fear, according to their efficacy beliefs. The model assumes people's actions are either “proactive, offensive, and engaged for danger control,” or “defensive, protective, and avoidance-driven for fear control” (Miles et al., 2008, p. 1873). As for the risk perception, people assess both the extent to which an existing risk is seen to be serious and about how vulnerable they are to the existing threat (Witte, 1994), which is called perceived severity and perceived susceptibility (Sheeran et al., 2014). Previous studies indicate higher levels of perceived severity and susceptibility motivate people to take protective actions—for example, by seeking updates about emergencies and following instructions from authorities (Sheeran et al., 2014; McCaughey et al., 2018).

Studies of risk-related information behaviour demonstrate a positive relationship between individuals' risk perception and information-seeking behaviour (Gutteling and de Vries, 2017; Deline and Kahlor, 2019). Whereas multiple studies indicated that risk perception increases information-seeking behaviours, the relationship between risk perception and information avoidance appears more complex. Some found risk perception positively predicts information-seeking and -avoidance behaviours (Witte et al., 1996; Taber et al., 2015). Others found risk perception positively predicts information-seeking but negatively predicts information avoidance (Yang and Kahlor, 2013). The lack of consensus suggests individuals' risk perceptions may influence information-related behaviour, especially information-avoidance behaviour, in a complex way. To investigate the reasons for differential effects on information-seeking and -avoidance behaviours during an acute risk, this research will separately examine both aspects of risk perception: perceived severity and perceived susceptibility.

#### Perceived Efficacy

Perceived efficacy is an individual's evaluation of effectiveness, feasibility, and convenience in the face of a threat (Sweeny et al., 2014). Based on their appraisal of the efficacy, they often chose either protective or defensive strategies to cope with severe threats. Previous research indicates that perceived risk and efficacy positively predict risk information-seeking behaviour (Kievik and Gutteling, 2011). However, in situations where the threat exceeds people's perceived efficacy, people may choose not to control the threat. Instead, they defensively control their emotions, like fear, with avoidance-based strategies such as denying the need to act and the existence of danger (Li, 2018). In this study, we may characterise peoples' inattentiveness to vital information during a crisis as defensive, avoidant behaviour (Miles et al., 2008). Perceived efficacy for risks includes both self-efficacy and response efficacy. Self-efficacy is a person's perception of his/her ability to implement the recommended response suggested by governors, professionals or to reduce the threat. Furthermore, response efficacy refers to a person's belief in the effectiveness of the recommended response in stopping the threat (Witte, 1998). To investigate how perceived efficacy for risk correlates with information behaviours differently, the effects of efficacy for self and response on information behaviours were examined separately.

### Affective Factors That Relates to Information-Related Behaviour

Gutteling and de Vries (2017) assert affective responses to perceived risks make people more aware of their personal relevance to threats. However, the empirical evidence on how affective factors are related to information-related behaviour is inconsistent and fragmented. Most studies focus on one or two affective factors, such as anger or feeling worried (Griffin et al., 1999, 2008; Yang et al., 2014) or sadness and happiness (Tiedens and Linton, 2001). Scholars proposed that risk perception is directly related to the native valence of affects, as threats are likely to produce negative emotions (Griffin et al., 1999); however, perceived threats can produce positive affective responses as well (Griffin et al., 2008). Empirical evidence suggests that inconclusive evaluations of threats can promote systematic information-processing (Tiedens and Linton, 2001), and possibly motivate proactive information seeking.

These researches have indicated that both negative and positive emotions can stimulate information-seeking behaviour, especially in high-risk contexts. Therefore, the relationship between risk perception and information-related behaviour may be context-specific and dependent on individual preferences. Based on the positive and negative valence of affect, Yang examined affective responses to risk by measuring several specific affective factors (Yang, 2012; Yang and Kahlor, 2013). They found peoples' negative emotions regarding climate change stimulate information-seeking behaviour, and peoples' optimism about the same issue led to information-avoidance behaviour. This result needs to be interpreted in the context of chronic risks, where people may not perceive a strong sense of urgency or prioritise acting immediately. However, whether the finding could be applied to acute risk situations should be examined and discussed, as high levels of urgency and threat under such situations may cause avoidance from the discomfort of negative feelings. To investigate how different affective responses correlates with information-seeking and -avoidance behaviour, six affective responses about the issue of the COVID-19 pandemic were examined.

**Figure 1:**
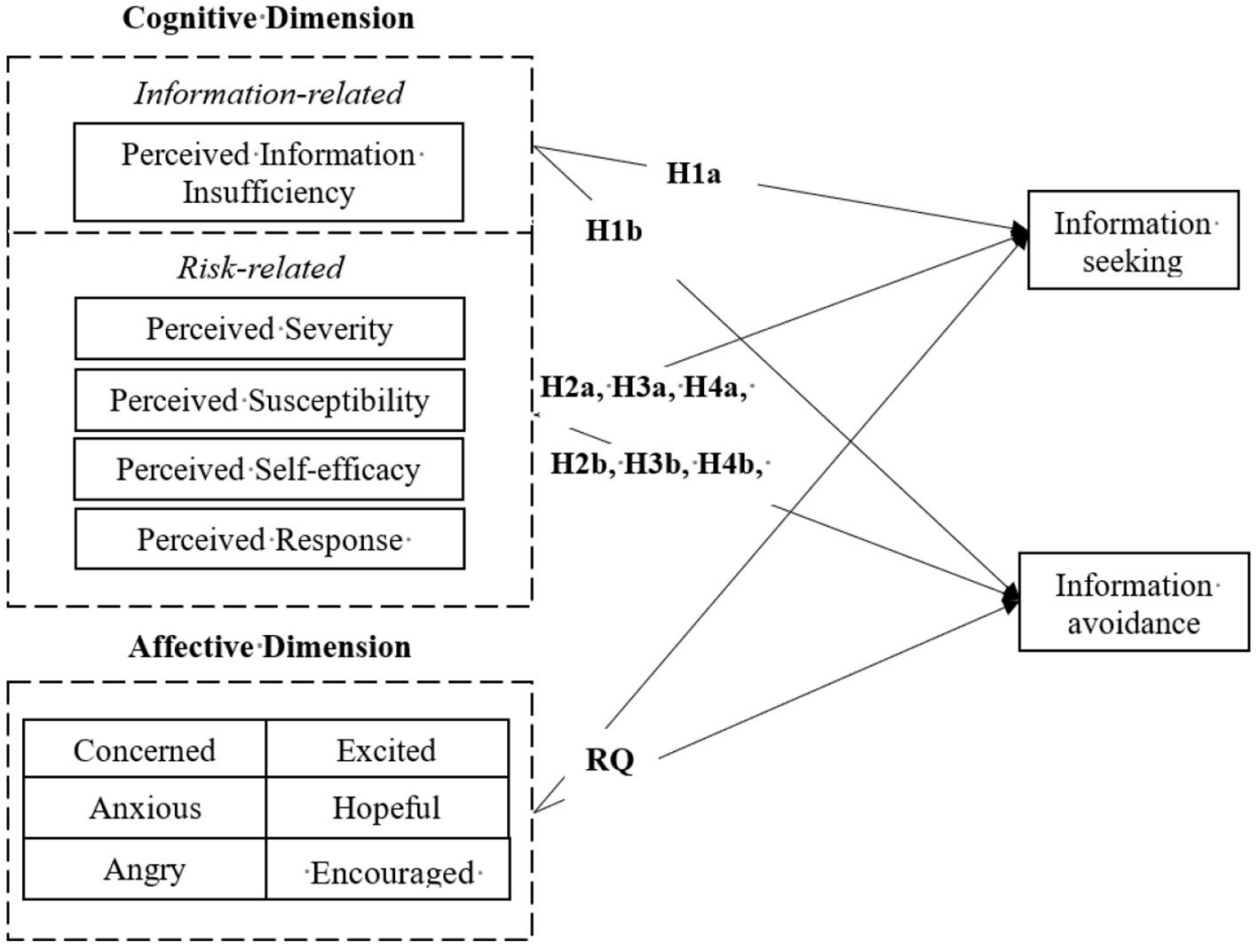
The cognitive and affective predictors on information-seeking and information-avoidance behaviour.

We developed the following five hypotheses regarding how cognitive factors are related to information-seeking or -avoidance behaviour under acute risks. We also identified a research question to explore how affective responses to risks correlates with information behaviour under acute risks (see in Figure 1).

Hypothesis 1: Individuals' perceived level of perceived information insufficiency towards COVID-19 will be: a) positively related to information-seeking behaviour, and b) negatively related to information-avoidance behaviour.
Hypothesis 2: Individuals' perceptions of the severity of COVID-19 will be positively related to a) information-seeking, and b) information-avoidance behaviour.
Hypothesis 3: Individuals' perception of their susceptibility for COVID-19 will be positively related to a) information-seeking, and b) information-avoidance behaviour.
Hypothesis 4: Individuals' perceived self-efficacy regarding COVID-19 prevention will be: a) positively related to information-seeking behaviour, and b) negatively related to information-avoidance behaviour.
Hypothesis 5: Individuals' perceptions of their response efficacy towards COVID-19 prevention will be: (a) positively related to information-seeking behaviour, and (b) negatively related to information-avoidance behaviour.
Research Question: How are peoples' different affective responses related to information-seeking and information-avoidance behaviour?

## Methods

### Data Collection

Data were collected through a survey of Chinese residents between February 25 and 28, 2020. We employed a quota sampling method based on China's population distribution by province. The survey's URL link or QR code was sent to prospective respondents through social networking platforms. This study was reviewed and approved by School of Journalism and Communication, Nanjing University. A cover page told participants that they would take part in a research about health-related behaviour. All provided informed consent before enrolling and completing the survey. The population comprised Chinese residents aged 18 years and above who had Internet access. Because of budget and resource constraints, the target sample size was 2,000 people; after excluding invalid responses, the final valid sample size was 1,946 people. Among the respondents, 63% were male and 37% were female compared to 51.2 vs. 48.8% in the population. Of these, 90.4% were 18–40 years old, and 68.6% holds a bachelor's degree or higher (compared to 15.5% in population). For other variables using multiple-item scales, we used their their mean value of all items as the score.

### Measures

#### Perceived Information Insufficiency

Perceived information insufficiency gauges the gap that exists between the perceived current knowledge and the information sufficiency threshold (Griffin et al., 2004a).

They measured participants' perceptions of their current knowledge and sufficiency threshold on a scale ranging from 0 to 100. In this research, participants were asked to rate their current knowledge of COVID-19 and the amount of information they felt would be sufficient for them to appropriately confront the pandemic. We subtracted the former score from the latter to measure participants' perceived information insufficiency (mean = −5.91, standard deviation or *SD* = 29.63).

#### Perceived Severity and Perceived Susceptibility

Following the Risk Behaviour Diagnosis Scale of Witte and colleagues, we measured participants' perception of threats on a six-item scale (Witte et al., 1996). It measured perceived severity and perceived susceptibility using three items for each. We modified the general threat-related statements to ask specific questions about participants' COVID-19 threat perception. They were asked to indicate their level of agreement with the survey's statements on a seven-point Likert scale, where 1 = “*completely disagree*” and 7 = “*completely agree*.” The statement measuring perceived severity was, “I believe that the pandemic is severe/serious/significant” (mean = 5.06, *SD* = 1.47, Cronbach's α = 0.88); and the statement measuring perceived susceptibility was, “I am at the risk of / It is likely that I will contract / It is possible that I will contract COVID-19” (mean = 3.21, *SD* = 1.60, Cronbach's α = 0.87).

#### Perceived Self-Efficacy and Perceived Response Efficacy

We measured perceived efficacy by a scale established by Witte and colleagues—i.e., a two-dimensional, six-item scale using the same response scale of perceived threats, where the two dimensions were self-efficacy and response efficacy (Witte et al., 1996). In this study, the recommended response was washing one's hands and wearing a face mask in public. Statements used to measure response efficacy were, “The recommended response works to prevent COVID-19 disease,” “The recommended response effectively prevents COVID-19 disease,” and “If I respond in the recommended way, I am less likely to get COVID-19 disease” (mean = 5.71, *SD* = 1.24, Cronbach's α = 0.88). Statements used to measure self-efficacy were, “I am able to respond in the recommended way in order to prevent myself from getting COVID-19 disease,” “The recommended response is easy to do,” and “The recommended response is convenient” (mean = 5.72, *SD* = 1.23, Cronbach's α = 0.87).

#### Affective Response

Following Yang and Kahlor, we measured participants' affective responses to the pandemic through six items composed of both positive and negative aspects (Yang and Kahlor, 2013). For positive affective factors, respondents were asked the extent to which they felt concerned (mean = 4.24, *SD* = 1.40), anxious (mean = 3.82, SD = 1.46), angry (mean = 3.42, *SD* = 1.58), excited (mean = 3.03, *SD* = 1.66), hopeful (mean = 4.78, *SD* = 1.26), and encouraged (mean = 4.11, SD = 1.52) about the pandemic, and their responses were registered on a six-point scale ranging from “*not at all*” (1) to “*very*” (6).

#### Information-Seeking

The five-item information-seeking scale developed by Griffin et al. (2004b) contains a reversed item that may confuse the respondents with information-avoidance behaviour. Therefore, we removed the reverse-coded item and asked participants to report their frequency of the following behaviours in the past month through a four-item, five-point frequency scale (from 1 = “*never*” to 5 = “*always*”). The four items were, “I actively search for pandemic-related information,” “I actively follow the latest pandemic information,” “I am likely to go out of my way to get more information about the pandemic,” and “I try to learn more information about the pandemic through various channels.” The reliability of this four-item scale was relatively high (mean = 3.59, *SD* = 0.90, Cronbach's α = 0.91).

#### Information-Avoidance

To measure information-avoidance behaviour, we adopted the scale developed by Yang and Kahlor (2013). The climate change topics of the original scale were adapted to pandemic-related ones. Participants responded to the following five items through a five-point scale (from 1 = “*never*” to 5 = “*always*”): “I avoid information about the pandemic,” “When it comes to the pandemic, I don't want to know more,” “I refuse to listen to information about the pandemic,” “I tune out information about the pandemic,” and “I ignore information about the pandemic” (mean = 2.31, *SD* = 1.11, Cronbach's α = 0.94).

#### Control Variables

Demographic variables were measured as control variables. Gender was coded as dummy variable (0 for female, 1 for male). Age was measure in three brackets (18–40, 41–60, beyond 60). Educational background was measured by asking the highest level of education (Primary, Junior high school, Senior High School, College, Bachelor, Master or above).

## Results

The descriptive analysis of all variables is shown in Table 1.

**Table 1:** Descriptive analysis of all variables.

**Variables**	**N (%)**	**Min**	**Max**	**Mean**	**SD**	**Cronbach's α**
Age						
18–40	1760 (90.4)					
41–60	182 (9.4)					
>60	4 (0.2)					
Level of Education						
Primary	21 (1.1)					
Junior High School	119 (6.1)					
Senior High School	471 (24.2)					
College	518 (26.6)					
Bachelor	749 (38.5)					
Master or above	68(3.5)					
Gender						
Male	1226 (63)					
Female	720 (37)					
Information seeking		1	5	3.59	0.9	0.91
Information avoidance		1	5	2.31	1.11	0.94
Information sufficiency		−100	100	−5.91	29.63	
Perceived severity		1	7	5.06	1.47	0.88
Perceived susceptibility		1	7	3.21	1.6	0.87
Self-efficacy		1	7	5.71	1.24	0.88
Response efficacy		1	7	5.72	1.23	0.87
Concerned		1	6	4.24	1.40	
Anxious		1	6	3.82	1.46	
Angry		1	6	3.42	1.58	
Excited		1	6	3.03	1.66	
Hopeful		1	6	4.78	1.26	
Encouraged		1	6	4.11	1.52	

The correlation matrix of the all variables is presented in Table 2.

**Table 2:** Zero-order correlation matrix of all variables.

	**Information avoidance**	**Perceived information insufficiency**	**Perceived severity**	**Perceived susceptibility**	**Self efficacy**	**Response efficacy**	**Concerned**	**Anxious**	**Angry**	**Excited**	**Hopeful**	**Encouraged**
Information seeking	−0.097^**^	−0.141^**^	0.323^**^	−0.046^*^	0.394^**^	0.393^**^	0.135^**^	0.074^**^	0.005	−0.059^**^	0.212^**^	0.149^**^
Information avoidance		0.176^**^	−0.118^**^	0.320^**^	−0.388^**^	−0.434^**^	0.031	0.146^**^	0.282^**^	0.325^**^	−0.111^**^	0.076^**^
Perceived information insufficiency			−0.083^**^	0.090^**^	−0.182^**^	−0.174^**^	−0.044	0.017	0.062^**^	0.109^**^	−0.076^**^	0.007
Perceived severity				0.090^**^	0.389^**^	0.402^**^	0.206^**^	0.150^**^	0.038	−0.099^**^	0.171^**^	0.083^**^
Perceived susceptibility					−0.323^**^	−0.310^**^	0.143^**^	0.229^**^	0.244^**^	0.261^**^	−0.117^**^	0.035
Self efficacy						0.868^**^	0.035	−0.090^**^	−0.199^**^	−0.257^**^	0.311^**^	0.103^**^
Responseefficacy							0.043	−0.087^**^	−0.218^**^	−0.285^**^	0.319^**^	0.089^**^
Concerned								0.619^**^	0.415^**^	0.014	0.067^**^	0.046^*^
Anxious									0.521^**^	0.166^**^	0.023	0.099^**^
Angry										0.299^**^	−0.041	0.115^**^
Excited											0.095^**^	0.369^**^
Hopeful												0.431^**^

** * Correlation is significant at the 0.01 level (2-tailed)*.

* * Correlation is significant at the 0.05 level (2-tailed)*.

To test the hypotheses, two hierarchical multiple regression models were built with information seeking and information avoidance as outcome variables separately (see Table 3). We entered demographic variables (age, gender, and education level) in the first block. Cognitive factors such as participants' perceptions of their perceived information insufficiency, risk severity, susceptibility, self-efficacy, and response efficacy were entered in the second block. The cognitive dimension factors of the second model accounted for 20% of the variance (Δ*R*^2^ = *0.1*9*, p*<*0.0*1) in information-seeking behaviour, and 21% of the variance in information-avoidance behaviour (ΔR^2^ = 0.25, *p* < 0.01). Then we entered the six affective dimension variables into the third block. The explanatory power of the third model became 21% in the regression model of information seeking (*R*^2^ = 0.21*, p*<*0.0*1), and 29% in the regression model of information avoidance (*R*^2^ = 0.29*, p* < 0.01).

**Table 3:** Hierarchical multiple regression effects on information-seeking and information-avoidance behaviour.

	**Information-seeking**			**Information-avoiding**		
	**Block 1**	**Block 2**	**Block 3**	**Block 1**	**Block 2**	**Block 3**
Gender	0.06^**^	0.02	0.02	−0.19^***^	−0.13^***^	−0.11^***^
Age	0.07^**^	0.04^*^	0.04	−0.01	−0.01	−0.04
Education	0.07^**^	0.00	0.01	−0.08^**^	−0.03	−0.02
Information insufficiency		−0.10^***^	−0.09^***^		0.04	0.05
Information insufficiency^2^		−0.05	−0.04		−0.08^**^	−0.05^*^
Perceived severity		0.17^***^	0.15^***^		0.02	0.01
Perceived susceptibility		0.06^*^	0.04		0.19^***^	0.14^***^
Self–efficacy		0.19^***^	0.18^***^		0.01	0.01
Response efficacy		0.17^***^	0.17^***^		−0.35^***^	−0.30^***^
Concerned			0.06^*^			−0.04
Anxious			0.02			0.02
Angry			0.02			0.14^***^
Excited			0.00			0.13^***^
Hopeful			0.04			−0.01
Encouraged			0.08^**^			0.03
**Adjusted R** ^**2**^	0.01^***^	0.2^***^	0.22^***^	0.04^***^	0.25^***^	0.29^***^
**R**^**2**^ **Change**		0.19^***^	0.02^***^		0.21^***^	0.04^***^

* * p <0.05*;

** * p <0.01*;

*** * p <0.001*.

Both hypotheses 1a and 1b were not supported, as the results demonstrated opposite findings. Contrary to hypothesis 1a, participants' perceived information insufficiency was negatively related to their information-seeking behaviour (β = −0.09*, p* < 0.01). Hypothesis 1b was not supported, since the relationship between perceived information insufficiency and information-avoidance behaviour (β = 0.05*, p* > 0.05). Moreover, a quadratic regression analysis was performed to quantify the relationship between information insufficiency and their corresponding information seeking and avoidance behaviour. The results showed that the squared term of information insufficiency is not significantly related to information seeking behaviour (β = −0.04*, p* > 0.05) and was negatively related to information avoidance behaviour (β = −0.05, *p* < 0.05). The regression equation was found to be: estimated information avoidance = 2.412 + 0.003(information insufficiency) −0.00009 (information insufficiency^2^). There indicates inverted “U-shape” relationship between information insufficiency and information avoidance behaviour.

Hypothesis 2a was supported, as participants' perceived risk severity positively predicted their information-seeking behaviour (β = 0.15*, p* < 0.001). Despite the positive effect of perceived severity on information-seeking, its effects on information avoidance behaviour were found to be insignificant (β = 0.01*, p* > 0.05), and thus hypothesis 2b was not supported.

Hypothesis 3a was not supported, while hypothesis 3b was supported; the effect of participants' perceived susceptibility turned out to be insignificant (β = 0.04*, p* > 0.05) after the affective factor variables were entered into the regression model. By contrast, the regression results showed that perceived susceptibility positively predicted information avoidance (β = 0.14*, p* < 0.001). Results for hypotheses 2 and 3 showed that two aspects of risk perception exerted a differentiated effect on information-seeking and -avoidance behaviour during COVID-19. Perceived severity only positively predicted information seeking (β = 0.15*, p* < 0.001), and perceived susceptibility positively predicted information avoidance (β = 0.14*, p* < 0.001).

Hypothesis 4a was supported since participants' perceived self-efficacy positively predicted information-seeking behaviour (β = 0.18*, p* < 0.001). However, hypothesis 4b was not supported, as the relationship between self-efficacy and information-avoidance behaviour was insignificant (β = 0.01*, p* > 0.05).

Both hypotheses 5a and 5b were supported, as participants' response efficacy had a positive effect on information-seeking behaviour (β = 0.17*, p* < 0.05) and a negative effect on information-avoidance behaviour (β = −0.30*, p* < 0.001).

Finally, we tested the research question about the effects of affective factors by entering the six affect-related variables into the third step of the hierarchical multiple linear regression model. The final model explained 22% of the variance in information-seeking behaviour and 29% of the variance in information-avoidance behaviour. Among the six affective factors, feeling concerned (β = 0.06, *p* < 0.05) and encouraged (β = 0.08, *p* < 0.01) were positively and significantly related to information-seeking behaviour. Other affective responses did not display a significant effect on information-seeking behaviour. Also, being angry (β = 0.14, *p* < 0.001) or excited about the pandemic (β = 0.13, *p* < 0.001) were positively related to participants' information-avoidance behaviour.

Table 2 displays the regression effects.

## Discussion

This paper examined the relationship between various cognitive factors and individuals' information-seeking and information-avoidance behaviours under the acute health risk of the COVID-19 pandemic. The results suggest that people's information behaviour under acute risks did not follow the sufficiency principle. Instead, perceived information insufficiency encourages information avoidance behaviour and discourages information-seeking behaviour. The findings provided support for the fear-control principle that predicts risk information behaviours, while it also demonstrated how the cognitive factors concerning fear-appraisal and response-appraisal stimulate information-seeking and information-avoidance behaviours in different ways.

### Cognitive Dimension

Perceived information insufficiency negatively correlates to information-seeking behaviour. In the COVID-19 context, the gap between peoples' actual and desired knowledge dulled their desire to actively seek pandemic-related information-seeking. There was an inverted U-shaped relationship between information insufficiency and avoidance behaviour. When the level of information insufficiency is relatively low, people deliberately avoid relevant information. When the gap of information inadequacy widens, they shift to a reduced tendency to avoid. These results were contrary to the Heuristic Systematic Model and its sufficiency principle proposed by Eagly and Chaiken (1993). We might explain this result by arguing that the information sufficiency principle, reliant on cost-benefit analysis and does not take into account the fact that more information does not always help people make informed decisions, especially in the Internet age. Indeed, more knowledge may cause cognitive dissonance (Festinger, 1962) or fear (Witte, 1994), especially among people with low judgmental confidence. Goodall and Reed (2013, p. 69) found people would rather maintain their uncertainty towards bed bug risk in their homes rather than know for sure that they are at risk; “individuals seek to maintain uncertainty, as it allows them to maintain their current state of information and avoid information that is likely to be distressing.” This cognitive process reveals that people often possess a defensive motive for personal beliefs, one that co-exists with their desire to hold an accurate belief (Chaiken et al., 1996).

In this study, risk-related cognitive factors had varying effects on information seeking and information-avoidance behaviour. In the COVID-19 context, individuals who perceived the situation as severe were more likely to actively seek information. However, when they saw a greater likelihood of being personally affected, they tend to avoid the information regarding the risks. Such differentiation between the effect of perceived severity and perceived personal susceptibility might account for the contradictory results of previous research that examined the impact of general risk perception on information-avoidance behaviour. This finding extends the impersonal impact hypothesis (Tyler and Cook, 1984), which held “media primarily increase societal-level risk perception, but they have little impact on personal-level risk perception,” (Oh et al., 2015; p. 15). El-Toukhy (2015) found individuals perceived different levels of susceptibility for themselves and others, which indicated an optimistic bias, while such a difference was not found in the perceived severity. In our study, the perceived severity of societal-level risks stimulated information-seeking behaviour and had little effect on information avoidance. By contrast, perceived susceptibility, as personal-level risk perception, stimulated information avoidance.

The results generally supported our hypothesis regarding self-efficacy, except the relationship between self-efficacy and information-avoidance behaviour was insignificant. These results were consistent with previous findings, which showed that when individuals perceived that recommended risk-prevention measures were feasible and effective, they were more willing to actively seek relevant information.

By contrast, higher perceptions of response efficacy could reduce information-avoidance behaviour. With COVID-19, if participants were confident the recommended public health measures could prevent the spread of the disease, they were more likely to engage in information-seeking behaviour. These results were consistent with the fear-control principles; namely, that perceived risks arouse people's fear, and that perceptions of efficacy determine risk responses. In this study, low levels of perceived response efficacy triggered a fear-control response and encouraged participants to deny or neglect a threat's severity, leading to information-avoidance behaviour. However, perceptions of self-efficacy did not trigger information-avoidance responses, possibly because perceptions of low efficacy may lead to fatalism (Miles et al., 2008).

### Affective Dimension

This study showed that the previous valence-based dichotomous classification of affective factors, where risk-related information had either a positive or negative effect on information behaviour, may not fully explain the information behaviour under acute-risk environments such as a global pandemic. Feeling concerned or encouraged positively relates to information-seeking behaviour, while information avoidance positively relates to feeling angry and excited. This demonstrated that the valence of affective responses cannot explain the differentiated effect that affective factors have on information behaviour. Su et al. (2021) found that the positive words (such as faith, blessing, praise, and love) on Chinese social media platform changed significantly across different stages of the pandemic. The use of positive emotive words, such as faith and blessing, indicates a concern for group cohesion and social solidarity during the outbreak of the epidemic. These results might explain why previous research findings were inconsistent. Our findings indicated that during a public health emergency, more intense and risk-heavy messages (such as angry and excited) may stimulate information-avoidance behaviour. This was contrary to a study that showed people's preference for attention to high-arousal messages (Lang et al., 1995).

This paper had several limitations. First, the results should be interpreted carefully with the consideration of the timing point of data collection, especially given the likelihood of recall and self-report bias. Due to the epidemic prevention and control policy at the time, we only conducted our research through the online channel, which to some extent made the sample more biassed towards the younger adult population. Also, due to the cross-sectional data design, all of the variables were measured simultaneously, which does not allow for establishing a causal relationship. Second, perceived information insufficiency was measured by calculating the difference between the two relevant components of insufficiency to simplify the results; other studies suggest taken different approaches (Kahlor et al., 2006; Griffin et al., 2008). Lastly, this research examined the direct impacts of the cognitive and affective antecedents of information behaviour, future research could examine how the factors interacts and goes beyond the addictive model.

## Conclusion

This paper makes several contributions to the literature on information-seeking and information-avoidance behaviours. First, it provided evidence that is contrary to the sufficiency principle predicting information behaviour in acute risks, suggesting the influence of other motives beyond the need for accuracy in acute risks. Second, differentiated effects of risk-perception-related variables on information-seeking and information avoidance should be noted. This demonstrated the necessity of further investigation into how personal- and society-level risk perceptions relates to fear-control responses leading to information-seeking or -avoidance behaviour. Third, by investigating various affective factors that information-related behaviour, this study asserted the aforementioned valence-based classification of affective factors may not clarify risk-related information behaviour. This study captures the information behaviour of individuals during acute and unknown risk outbreaks. In this case, people's behavioural rules may differ significantly from those of long-term, known, controlled risks. The COVID-19 pandemic will eventually be a thing of the past, but unknown, emergent risks are ever-present for humans.

## Data Availability Statement

The raw data supporting the conclusions of this article will be made available by the authors, without undue reservation.

## Ethics Statement

The studies involving human participants were reviewed and approved by School of Journalism and Communication, Nanjing University. The patients/participants provided their written informed consent to participate in this study.

## Author Contributions

SZ and YL contributed to conception and design of the study and wrote the first draft of the manuscript. SZ organized the database. YL performed the statistical analysis and wrote sections of the manuscript. Both authors contributed to manuscript revision, read, and approved the submitted version.

## Conflict of Interest

The authors declare that the research was conducted in the absence of any commercial or financial relationships that could be construed as a potential conflict of interest.

## Publisher's Note

All claims expressed in this article are solely those of the authors and do not necessarily represent those of their affiliated organizations, or those of the publisher, the editors and the reviewers. Any product that may be evaluated in this article, or claim that may be made by its manufacturer, is not guaranteed or endorsed by the publisher.
